# New Mitochondrial and Nuclear Evidences Support Recent Demographic Expansion and an Atypical Phylogeographic Pattern in the Spittlebug *Philaenus spumarius* (Hemiptera, Aphrophoridae)

**DOI:** 10.1371/journal.pone.0098375

**Published:** 2014-06-03

**Authors:** Ana S. B. Rodrigues, Sara E. Silva, Eduardo Marabuto, Diogo N. Silva, Mike R. Wilson, Vinton Thompson, Selçuk Yurtsever, Antti Halkka, Paulo A. V. Borges, José A. Quartau, Octávio S. Paulo, Sofia G. Seabra

**Affiliations:** 1 Computational Biology and Population Genomics Group, Centro de Biologia Ambiental, Departamento de Biologia Animal/Platform for Enhancing Ecological Research & Sustainability, Faculdade de Ciências, Universidade de Lisboa, Lisboa, Portugal; 2 Department of Natural Sciences, National Museum of Wales, Cardiff, United Kingdom; 3 Metropolitan College of New York, New York, New York, United States of America; 4 Biology Department, Science Faculty, Trakya University, Edirne, Turkey; 5 Department of Biological and Environmental Sciences, University of Helsinki, Helsinki, Finland; 6 Azorean Biodiversity Group, Centro de Investigação e Tecnologias Agrárias dos Açores and Platform for Enhancing Ecological Research & Sustainability, Universidade dos Açores, Departamento de Ciências Agrárias, Angra do Heroísmo, Terceira, Portugal; University of Innsbruck, AUSTRIA

## Abstract

*Philaenus spumarius* is a widespread insect species in the Holarctic region. Here, by focusing on the mtDNA gene COI but also using the COII and Cyt *b* genes and the nuclear gene EF-1α, we tried to explain how and when its current biogeographic pattern evolved by providing time estimates of the main demographic and evolutionary events and investigating its colonization patterns in and out of Eurasia. Evidence of recent divergence and expansion events at less than 0.5 Ma ago indicate that climate fluctuations in the Mid-Late Pleistocene were important in shaping the current phylogeographic pattern of the species. Data support a first split and differentiation of *P. spumarius* into two main mitochondrial lineages: the “western”, in the Mediterranean region and the “eastern”, in Anatolia/Caucasus. It also supports a following differentiation of the “western” lineage into two sub-lineages: the “western-Mediterranean”, in Iberia and the “eastern-Mediterranean” in the Balkans. The recent pattern seems to result from postglacial range expansion from Iberia and Caucasus/Anatolia, thus not following one of the four common paradigms. Unexpected patterns of recent gene-flow events between Mediterranean peninsulas, a close relationship between Iberia and North Africa, as well as high levels of genetic diversity being maintained in northern Europe were found. The mitochondrial pattern does not exactly match to the nuclear pattern suggesting that the current biogeographic pattern of *P. spumarius* may be the result of both secondary admixture and incomplete lineage sorting. The hypothesis of recent colonization of North America from both western and northern Europe is corroborated by our data and probably resulted from accidental human translocations. A probable British origin for the populations of the Azores and New Zealand was revealed, however, for the Azores the distribution of populations in high altitude native forests is somewhat puzzling and may imply a natural colonization of the archipelago.

## Introduction

Distribution patterns of animals and plants have faced dramatic changes throughout time and are influenced by ecological requirements and historical factors. In the northern hemisphere, Quaternary long-term glacial (cold) and interglacial (warm) climatic cycles that started about 2.6 million years (Ma) ago [1] have strongly influenced the species distributions and range sizes and, as a consequence, have affected the genetic structure of their populations [2], [3]. Evidence from numerous studies suggests that southern European regions of Iberia, Italy and the Balkans and areas near the Caucasus and western Asia, acted as glacial refugia for temperate species during cold periods [4], [5], [6]. Recent work indicates that temperate refugia might not have been restricted to the three southern peninsulas and that cryptic northern refugia might have existed in central, western, eastern and even northern Europe in the Late Pleistocene [7], [8], [9]. The relative impact of the post-glacial colonization history and more recent processes such as gene flow and population fluctuations, strongly depend on the dispersal mode and ability of the species [10], [11].

Genetic analyses have proven to be useful for a more detailed understanding of post-glacial expansions of several animals and plants [3], [12]. Mitochondrial DNA (mtDNA), due to its particular characteristics, has been widely used in determining population dynamics and phylogeographic divergence in recent times, such as the Quaternary period [13]. Nevertheless, the signal of deeper history can be obscured by homoplasy or saturation resulting from high mutation rate. On the other hand, reconstructing evolutionary histories using individual genes (gene trees) could lead to misrepresentation of population or species histories because in this case mtDNA, which reflects matrilineal history, might not represent the overall lineage history of the species. Also, if multiple population divergences or speciation events were closely spaced in time, a single gene tree might be ‘incorrect’ by chance due to the random nature of lineage sorting during the coalescence process [14]. Therefore, the use of multiple types of molecular markers is recommended.

Insects have been widely used as models for animal biogeographical studies (e.g., [15], [16], [17]). The meadow spittlebug *Philaenus spumarius* (Linnaeus, 1758) (Hemiptera, Aphrophoridae) is a widely investigated species, very suitable for genetic and ecological studies. It is a highly polyphagous insect which can be found in a variety of terrestrial plant communities and habitats, being the most common species within the genus *Philaenus* [18], [19]. It is widespread across the Palaearctic region from where it is native [20] having also colonized the Azores [21], [22] and has been introduced in the Nearctic region [20] and New Zealand [23]. The meadow spittlebug is very sensitive to humidity and temperature, especially in the earlier stages of its life cycle, which limits its range [24]. A remarkable example was reported for some North American populations where a northward range shift, probably as a result of climatic changes, was detected by [25]. This species shows a well studied dorsal colour polymorphism with eleven main described phenotypes which can be divided in melanic and non-melanic forms [20]. The phenotype frequencies differ among populations, probably due to the effects of natural selection under different habitats, climatic conditions and predation pressure (reviewed in [18], [19]). Recent studies on the genetic diversity of *P. spumarius* have given insights on its evolutionary history suggesting two routes of post-glacial colonization of higher latitudes in Europe and indicating a probable western European origin for North American populations [26], [27].

In the present study we tried to explain how and when the current biogeographic pattern of *P. spumarius* evolved by (i) providing time estimates of the main demographic and evolutionary events with focus on the populations occurring in the main Mediterranean peninsulas; and, (ii) investigating the colonization patterns out of Eurasia, namely of north-western Africa, North America, and the islands of the Azores and New Zealand.

## Material and Methods

### Ethics Statement

The field sampling was carried out on private lands with owners' permissions. The studied species, *Philaenus spumarius*, is considered a widespread species across the Palaearctic and the Nearctic regions, being a crop pest in some locations of USA and Canada. It is not an endangered or protected species.

### Sampling

**Figure 1:**
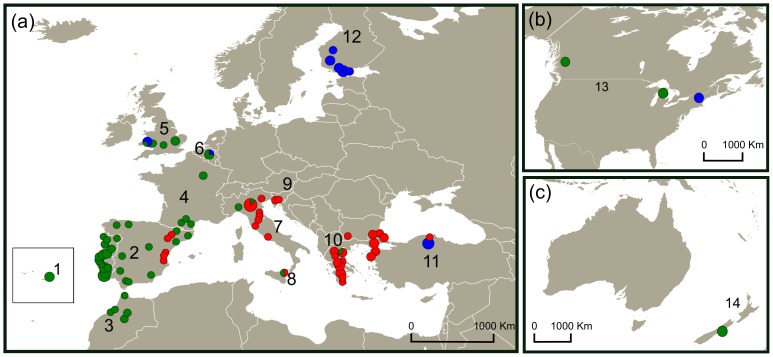
Sampling locations of *Philaenus spumarius* in (a) Europe and Anatolia (b) North America and (c) New Zealand in each geographic region. 1 – Azores; 2 – Iberian Peninsula; 3 – Morocco; 4 – France; 5 – United Kingdom; 6 – Belgium; 7 – Italian Peninsula; 8 – Sicily; 9 – Slovenia; 10 – Balkans (Bulgaria; Greece; European Turkey); 11 – Anatolian Peninsula; 12 – Finland; 13 – North America (Canada and United States of America); 14 – New Zealand. Circle sizes are proportional to the number of individuals. Circles: green – “western-Mediterranean” mtDNA group; red – “eastern-Mediterranean” mtDNA group; blue – “eastern” mtDNA group. Circle sizes are proportional to the number of samples.

A total of 196 specimens of *P. spumarius* were collected or sent by collaborators between 2007 and 2011 from 75 sampling locations across Europe, two from Anatolia, five from North Africa, three from North America and one from New Zealand (Fig. 1 and Table S1). Adult insects were captured using a sweep net suitable for low-growing vegetation and an entomological aspirator. In some cases, larval stages were collected by hand. Specimens were preserved in absolute ethanol or dried in silica gel and stored at room temperature.

### DNA extraction, amplification and sequencing

Entire larval stage specimens were used for DNA extraction while in the adults the wings and abdomen were removed and only the thorax and head were used. Genomic DNA was extracted using the E.Z.N.A. Tissue DNA Isolation kit (Omega Bio-Tek) and a 800 bp fragment of the 3'-end of the mitochondrial gene cytochrome *c* oxidase subunit I (COI) was amplified by polymerase chain reaction (PCR) using the primers C1-J-2195 (5′–TTGATTTTTTGGTCATCCAGAAGT–3') and TL2-N-3014 (5′–TCCAATGCACTAATCTGCCATATTA–3') [28]. Due to DNA degradation in the New Zealand samples, a new set of primers, COI-PspF (5'–GTATAGATGTTGATACACGTGC–3') and COI-PspR (5'–TCCAGTAAATAAAGGGTATC–3') was designed to amplify an informative smaller fragment with 300 bp of COI that included the variable sites that differentiate the different haplogroups. Fragments with 500 bp of the mitochondrial genes cytochrome *c* oxidase subunit II (COII) and cytochrome *b* (cyt *b*) were also amplified using the primers TL2-J3033 (5'–GATATGGCAGAAATAGTGCA–3') and C2-N3665 (5'–CCACAAATTTCTGAACACTG–3') and CB-N3665 (5'–GTCCTACCATGAGGTCAAATATC–3') and CB-N11526 (5'–TTCAACTGGTCGTGCTCC–3'), respectively [29]. The three mitochondrial genes were sequenced initially in a subset of samples and similar genetic patterns and level of polymorphism were observed for the three mitochondrial genes on a preliminary analysis (results not shown). Thus, cytochrome *c* oxidase subunit I gene was sequenced for all individuals and cyt *b* and COII were only sequenced in a representative subset. The nuclear gene elongation factor-1α (EF-1α) is widely used in insect genetic studies. Therefore, we chose to sequence it in a subset of individuals that covered all the geographical areas of the study. A 700 bp fragment of the nuclear gene EF-1α was amplified using the primers M3 (5′–CACATYAACATTGTCGTSATYGG–3') and rcM44.9 (5′–CTTGATGAAATCYCTGTGTCC–3') [30]. For COI gene, PCR was performed in a 12.5 µL reaction volume containing: 1 µM of each primer, 0.1 mM dNTPs, 1 mM MgCl_2_, 2.5 µL 5x Colorless GoTaq Flexi Buffer, 0.02U GoTaq DNA Polimerase (Promega) and approximately 30 ng of DNA. The PCR conditions were: an initial denaturation step at 94 °C for 3 min, followed by 35 cycles of denaturation at 94 °C for 30 sec, annealing at 50 °C for 45 sec and extension at 72 °C for 1 min, with a final extension period at 72 °C for 7 min. The same PCR conditions were used for COII and cyt *b* genes except for annealing temperature where a touch up between 52.5 °C and 56 °C for COII and between 47 °C and 54 °C for cyt *b* was performed. Nuclear EF-1α gene PCR was performed in a 20 µL reaction volume containing: 0.6 µM of each primer, 0.2 mM dNTPs, 1.125 mM MgCl_2_, 0.8 µL BSA (10 µg/mL), 4.0 µL 5x Colorless GoTaq Flexi Buffer, 0.05U GoTaq DNA Polimerase (Promega) and approximately 30 ng of DNA. PCR conditions used were: an initial denaturation step at 95 °C for 5 min, followed by 40 cycles of denaturation at 95 °C for 45 sec, annealing at 59 °C for 35 sec and extension at 72 °C for 1 min, with a final extension period at 72 °C for 10 min. All PCR products were purified with SureClean (Bioline) following the manufacturer's protocol, sequenced using the forward and the reverse primers with the BigDye Terminator v3.1 Cycle Sequencing Kit (Applied Biosystems) and analysed on a genetic analyser ABI PRISM 310 (Applied Biosystems).

### Molecular and population structure analyses

Sequences were verified and edited using the software Sequencher v. 4.0.5 (Gene Codes Corporation) and BioEdit v. 7.0.9 [31]. They were then aligned using Mafft v. 7.029b (http://mafft.cbrc.jp/alignment/software/) and converted in the appropriate format with Concatenator v. 1.1.0 [32]. For nuclear EF-1α sequences, haplotype phase from heterozygous individuals for base positions and length-variable regions was determined using Champuru v. 1.0 [33]. Phylogenetic analysis using the Maximum Parsimony (MP), Maximum Likelihood (ML) and the Bayesian inference (BI) methods were performed for concatenated mtDNA genes (COI, COII and cyt *b*) and for the nuclear gene EF-1α in Paup v. 4.0.d99 [34] and in MrBayes v. 3.1.2 [35]. For MP and ML analysis a heuristic search was performed using 100 replicates and branch support was obtained by performing 1000 replicates of non-parametric bootstrap. Gaps were treated as a fifth base in MP. The BI analysis was performed using the Monte Carlo Markov Chain (MCMC) method iterated for 2 000 000 generations, with a sampling frequency of 1500 generations and a burn-in of 1000. For each dataset the best fit model of sequence evolution was estimated using Modeltest v. 3.7 [36] under the Akaike information criterion (AIC). Elongation factor-1α sequences of *P. spumarius* and *P. italosignus* from [26] and available at NCBI Genbank were added to our nuclear matrix and included in the phylogenetic analysis (GenBank accession numbers: JF309079 and JF309081-JF309095). *Philaenus italosignus* was used as outgroup in all phylogenetic analysis. Polymorphic sites and mtDNA haplotypes for COI, COII and cyt *b* genes were calculated using Mega v. 5.0 [37] and a median-joining haplotype network was constructed using Network v. 4.5.0.1 (Fluxus Technology Ltd. 2004). For COI mtDNA gene, haplotype (*h*) and nucleotide diversities (π) were calculated for each geographical region (defined as numbers in Fig. 1) and an analysis of molecular variance (AMOVA) was performed using Arlequin v. 3.5 [38] to assess population genetic structure of *P. spumarius*. The groupings were based in the several sub-regions of Europe, America, Africa and Asia. This analysis produces estimates of variance components and *F*-statistic analogues, designated as Ф-statistics, reflecting the correlation of haplotypes at different levels of hierarchical subdivision. Groupings with the highest significant Ф_CT_ value in AMOVA should reflect the most probable geographical subdivisions [39].

### Divergence time estimates

We used the software package Beast v. 1.7.0 [40] and the mtDNA gene COI to estimate divergence times of nodes of interest, as well as their demographic history via Bayesian Skyline plots (BSPs). For each dataset the best fit model of sequence evolution was estimated using Modeltest v. 3.7 under the Akaike information criterion (AIC) and a piece-wise constant Bayesian skyline tree prior was selected with 10 groups. Two additional analyses of our data with 5 and 15 groups to assess the impact of the number of groups on the final result were conducted. These analyses did not reveal a significant impact on the overall result, whether on the shape of the Bayesian Skyline plot or on the estimation of divergence times. Preliminary runs using the uncorrelated lognormal relaxed clock revealed a posterior distribution of the σ_r_ (“CoefficientOfVariation”) parameter consistently abutting 0, suggesting that the COI partitions do not significantly deviate from a strict clock assumption. Therefore, we employed a strict molecular clock for each dataset with a normal prior distribution on the substitution rate with a mean of 0.0354, following the conserved rate of 3.54% per million years as suggested by [41], and a standard deviation of 0.005 to account for rate uncertainty. Two Markov chains Monte Carlo (MCMC) of 50 000 000 generations, sampled at every 5000^th^ iteration, were conducted and combined with LogCombiner v. 1.6.1 [40]. Tracer v. 1.4 [42] was used to assess the convergence and mixing for all model parameters and to create the Bayesian Skyline plots.

### Demographic analyses and neutrality tests

Neutrality tests of Tajima's *D* [43] and Fu's *F* statistics [44] were performed using Arlequin. These statistics are widely used with molecular data to detect changes in population size and/or estimating deviations from neutrality, assuming a constant population size at mutation-drift equilibrium. Thus, significant negative values of Tajima's *D* and Fu's *Fs* are considered to be evidence of expanding populations. Signatures of population expansion can also be detected through the frequency distribution of the number of pairwise differences between haplotypes and thus statistics based on the mismatch distribution and taking into account the Sudden Expansion Model [45] were also performed to detect and estimate the time of population growth. Estimated expansion values were obtained using Arlequin and graphics of frequency distribution using DnaSP v. 5 [46]. To test the observed mismatch distribution goodness-of-fit to the Sudden Expansion Model and to obtain confidence intervals around the estimated mode of mismatch distribution, 1000 permutation replicates were used [47]. Statistically significant differences between observed and expected distributions were evaluated with the sum of the square deviations (*SSD*) and Harpending's raggedness index (*hg*) [48], [49].

Timing of the demographic expansion as well as the 95% confidence interval for each mitochondrial haplogroup was estimated by converting the expansion time parameter τ, generated by Arlequin, to time (t) in years using the formula τ  =  2ut, where u is the mutation rate per nucleotide per year multiplied by sequence length (i.e. number of nucleotides), and t is the time since population expansion in years [45], [49]. We assumed a generation time of one year [24] and the conserved evolutionary rate of 3.54% per million years suggested by [41] for insect mitochondrial gene COI.

## Results

A total fragment of 539 bp was obtained for the mitochondrial gene COI in 190 samples. The remaining samples from Finland, Turkey, Canada (GenBank accession numbers: KJ699232–KJ699234) and New Zealand were not included in the main analysis due to their reduced size. In the 190 individuals there were a total of 71 haplotypes (GenBank accession numbers: KC111886 – KC111956) of which 44 occurred only once (Table S2). Of a total of 539 sites sequenced, 53 were polymorphic but only 26 were parsimony informative. For the mtDNA COII (495bp) and cyt *b* (434bp) genes, 47 individuals were sequenced and a total of 14 (GenBank accession numbers: KF280589 – KF280602) and 18 haplotypes (GenBank accession numbers: KF280603 – KF280620) were found, respectively. As commonly observed for insects [28], nucleotide sequences were A+T rich (approximately 71%). No gaps or early stop codons were detected in the 3 mtDNA genes sequences suggesting that all of them are functional mitochondrial DNA copies.

From the 24 individuals sequenced, we were able to successfully sequence a fragment of the nuclear gene EF-1α for only 13 individuals of *P. spumarius*. Almost all sequences exhibited double peaks due to the frameshift resulting from indels (length-variable regions) located at several sites of the intron in the EF-1α gene. Ten individuals were heterozygous in respect to indels and/or to base positions and the phased haplotypes (*alleles*) were differentiated by adding the letter *a* or *b* at the end of name (Table S1) (GenBank accession numbers: KF280621 – KF280642).

### Phylogenetic and population structure analyses

**Figure 2:**
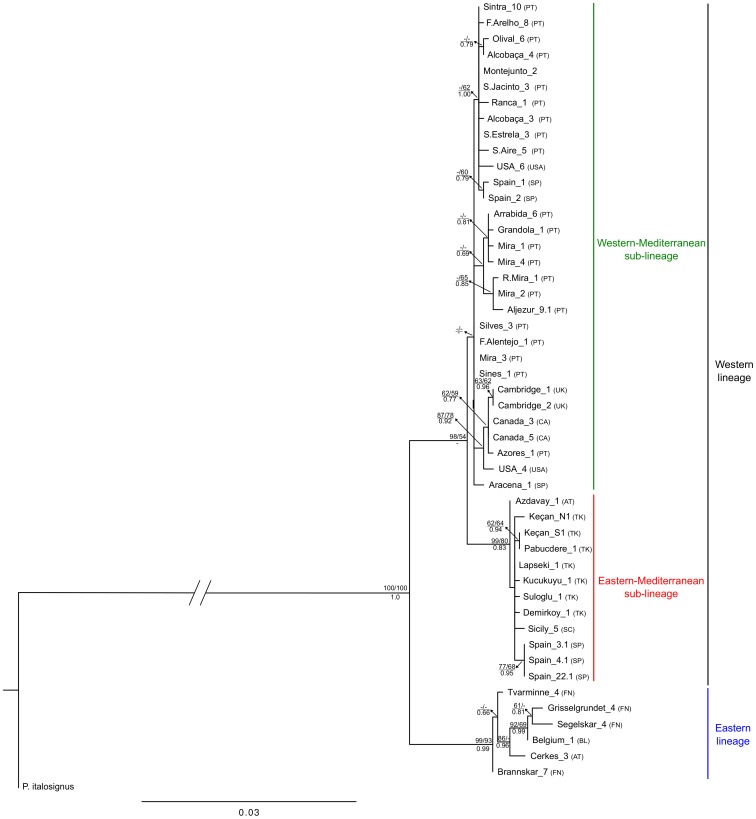
Maximum Likelihood tree based on the 3 concatenated mtDNA genes (COI, COII and cyt *b*) (1527bp). Values above branches correspond to MP and ML bootstrap values (only values > 50% are shown) and values below branches correspond to Bayesian posterior probability. PT – Portugal; SP – Spain; UK – United Kingdom; BL – Belgium; FN – Finland; SC – Sicily; TK – European Turkey; AT – Anatolia; USA – United States of America; CA – Canada.

**Figure 3:**
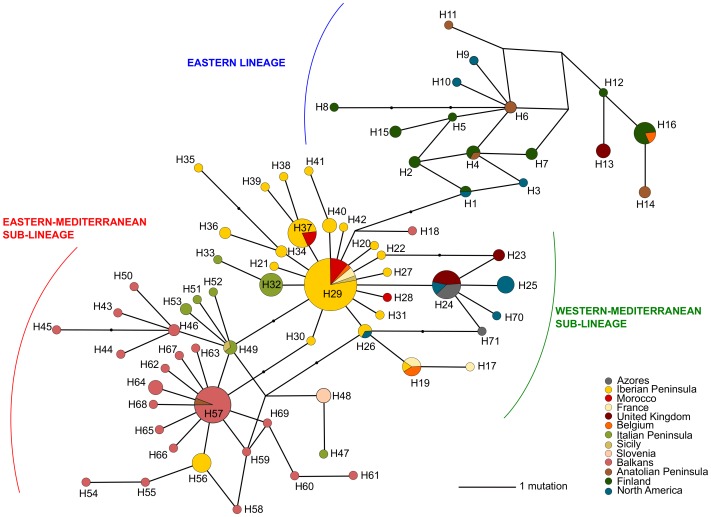
Median-joining haplotype network of *Philaenus spumarius* sampled geographic regions for mitochondrial gene COI (539bp). Size of the circles is in proportion to the number of haplotypes. Branches begin in the centre of the circles and their sizes are in proportion to the number of mutations.

Phylogenetic trees obtained for concatenated mtDNA genes and for a subset of *P. spumarius* individuals by the three methods, MP, ML and BI, presented a congruent topology. Maximum likelihood (Fig. 2), MP (not shown) and BI (not shown) trees, revealed the existence of two main haplotype groups: the “western” and the “eastern”. The “western” is divided in the “western-Mediterranean” and the “eastern-Mediterranean” sub-groups. The same phylogeographic pattern was found in the COI median-joining haplotype network (Fig. 3) and also in the COII and Cyt *b* median-joining haplotype networks (Fig. S1 and S2). The “eastern” haplogroup includes haplotypes from a wide geographical area, including northern Anatolia (Cerkes), Finland, Belgium, the UK (Aberdare – Wales) and eastern North America (New Hampshire – USA). In the “western-Mediterranean” group, the most common haplotype (H29) and several derived haplotypes, differing by one or two mutational steps, are shared between populations from the Iberian Peninsula, Morocco, France, Belgium, Italian Peninsula, Sicily and one individual from Balkans (H18). A group of haplotypes derived from H29 (H23, H24, H25, H70 and H71) includes samples from the Azores, western North America (British Columbia – Canada), eastern North America (Michigan – USA) and the UK, differing by two or three mutations. In the “eastern-Mediterranean” group, a similar star-like pattern is present with rare haplotypes connected to the most common (H57), usually by one mutational step. This group encompasses populations from the Balkans (Greece, Bulgaria and European Turkey), Slovenia, Italian Peninsula and Sicily. This lineage is also present in five samples from the eastern part of the Iberian Peninsula (H56) (Figs. 1 and 2).

The haplotype median-joining network based on smaller sequences of 289 bp of the COI gene, used in this analysis to include the samples from New Zealand, showed a total of 31 haplotypes and a pattern congruent to that observed for the 539 bp of COI region, with the same three distinct groups (“western-Mediterranean”, “eastern-Mediterranean” and “eastern”). The haplotype H14 belonging to the “western” haplogroup was found in the three New Zealand individuals and this same haplotype was shared with seven individuals from the UK, five from the Azores, three from Canada and four from the USA (Fig. S3).

**Figure 4:**
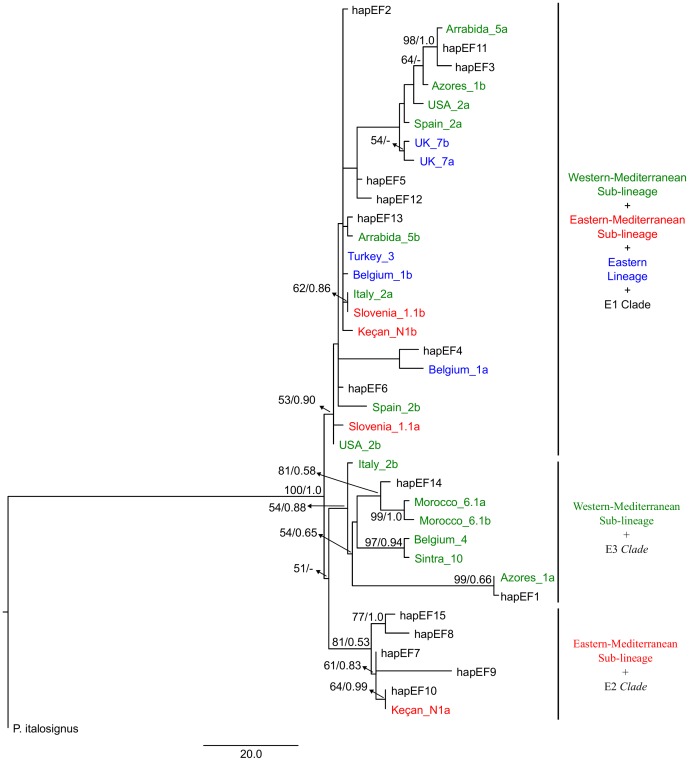
Maximum Parsimony tree based on nuclear gene elongation factor-1α. Values above branches correspond to MP bootstrap (only values > 50% are shown) and Bayesian posterior probability values. Black: GenBank sequences (see [26]); blue individuals correspond to the eastern mtDNA group; red individuals correspond to the eastern-Mediterranean mtDNA group and green individuals to the western-Mediterranean mtDNA group.

In the MP (Fig. 4) and BI (not shown) phylogenetic trees obtained for nuclear gene EF-1α three main groups could also be distinguished: *clade* A, *clade* B and *clade* C. However, these groups were not totally congruent to the groups found for the concatenated mtDNA genes. The *clade* A includes samples from our “eastern-Mediterranean” haplogroup and also from Georgia, Bulgaria, Hungary, Greece and Italy (E2 *clade*, in [26]). The *clade* B includes individuals from our “western-Mediterranean” group and from Portugal, Spain and Italy (E3 *clade*, in [26]). The *clade* C, however, is constituted by individuals from our three main mitochondrial groups and from Russia, Norway, Alps, Crimea, Poland and Ukraine (E1 *clade*, in [26]). Although the three groups have good bootstrap support, the branching order is unsolved since there is very low support for the branch clustering the *clades* A and B (Fig. 4). We also observed that both alleles of the most heterozygous samples are clustered within the same *clade* (*Clade* C – Arrabida_5, Spain_2, UK_7, Belgium_1, Slovenia_1.1 and USA_2 and *Clade* B – Morocco_6.1), with the exception for the Azores_1, Italy_2 and Keçan_N1 samples which have one allele in the *clade* C and the other allele in the *clades* A or B.

### Genetic variability and diversity

Mitochondrial haplotype diversity of COI was generally high (*h* > 0.6000), except for the Azores (*h*  =  0.4000) and Slovenia (*h*  =  0.0000) (Table 1), that may be related to the low sample size. On the other hand, Anatolia and the Balkans have the highest values of haplotype diversity (*h*  =  0.9048 and *h*  =  0.9128, respectively). Interestingly, Finland and North America also have high haplotype diversity (*h*  =  0.8971 and *h*  =  0.8939). Analysing nucleotide diversity, Anatolia, United Kingdom and North America have the highest values (π  =  0.009895, π  =  0.009318 and π  =  0.008236, respectively). The high values of both haplotype and nucleotide diversities detected in Anatolia and North America are likely a result of the presence of different mitochondrial lineages in these regions. The AMOVA performed for the *P. spumarius* groupings defined revealed that most of the genetic variation (47% and 43.87%) is explained by genetic differences within populations and not by geographic subdivisions (Table S3).

**Table 1:** Number of individuals, number of haplotypes and genetic diversity indices calculated for geographic regions of *Philaenus spumarius* and for mitochondrial gene Cytochrome *c* oxidase I (COI).

Geographic regions	Number of	Number of	Haplotype diversity (*h*)	Nucleotide diversity (π)
	individuals	haplotypes		
Morocco	7	3	0.6667 +/− 0.1598	0.001414 +/− 0.001338
Iberian Peninsula	63	19	0.7798 +/− 0.0493	0.003133 +/− 0.002062
Azores	5	2	0.4000 +/− 0.2373	0.000742 +/− 0.000944
Western Europe	9	4	0.7500 +/− 0.1121	0.006597 +/− 0.004194
Slovenia	3	1	0.0000 +/− 0.0000	0.000000 +/− 0.000000
Italy	17	8	0.8162 +/− 0.0815	0.004666 +/− 0.002957
Balkans	40	21	0.9128 +/− 0.0303	0.003825 +/− 0.002431
Anatolian Peninsula	7	5	0.9048 +/− 0.1033	0.009895 +/− 0.006223
United Kingdom	10	3	0.6889 +/− 0.1038	0.009318 +/− 0.005592
Finland	17	9	0.8971 +/− 0.0534	0.006603 +/− 0.003950
North America	12	8	0.8939 +/− 0.0777	0.008236 +/− 0.004917

Western Europe: Belgium and France; Italy: Italian peninsula and Sicily.

### Divergence times

The mean ages and 95% highest posterior density (HPD) determined for the TMRCA of mtDNA COI haplogroups are presented in Table S4. Estimated divergence times of all mitochondrial groups are less than 0.5 Ma. The “eastern” mtDNA group seems to be the oldest having diverged from the “western” mtDNA group at approximately 0.270 Ma ago and then begun its diversification around 0.190 Ma ago (0.374–0.056 Ma ago). The split of the “western” mtDNA group into the “eastern-Mediterranean” and the “western-Mediterranean” sub-groups was estimated to have occurred at approximately 0.146 Ma ago (0.243–0.067 Ma ago), while the TMRCA of both groups was quite similar and was estimated to be around 0.080 Ma ago. The confidence interval associated with our time estimates, however, is broad and the mutation rate of 3.54% per million years, in which these calculations are based, was estimated for COI in tenebrionid beetles [41]. Therefore, our results should be treated with caution and regarded as the best approximations given the current methods and calibrations [50], [51].

### Demographic analyses and neutrality tests

The demographic history of *P. spumarius* populations was analysed separately for the three COI groups. The distribution of pairwise nucleotide differences (mismatch distribution) showed that the “western-Mediterranean” and the “eastern-Mediterranean” groups exhibited a smooth and unimodal shape while the “eastern” group revealed a slightly bimodal curve (Fig. S4). All distributions, except for the “eastern” group distribution, were consistent with sudden and spatial population expansions. The observed raggedness index was low for all groups and both *P*_SSD_ and *P*_RAG_ showed that the observed distributions did not differ significantly from those expected under a sudden and a spatial population expansion model (Table 2). Negative significant deviations from neutrality were detected with Tajima's *D* and Fu's *F* statistics for the “western-Mediterranean” and the “eastern-Mediterranean” mtDNA groups, which corroborate the hypothesis of past population expansion events. The “eastern” group presented a non significant *p*-value with Tajima's *D* test although the Fu's *F* was significant (Table 3) indicating that it may have undergone negligible population growth. Demographic reconstructions (BSPs) for all mtDNA groups suggest a trend of population growth (Fig. S5), with a more evident demographic expansion in the “western-Mediterranean” and the “eastern-Mediterranean” mtDNA groups, and very slight or absent population growth for the “eastern” lineage.

**Table 2:** Parameters from the mismatch distribution for *Philaenus spumarius* COI groups.

	Mismatch Analysis						
	Demographic Expansion						
	Parameters						
	θ_0_ (CI = 95%)	θ_1_ (CI = 95%)	**τ** (CI = 95%)	SSD	P_SSD_	Raggedness	P_rag_
Eastern Group	0.00176 (0.000–1.366)	14.41895 (8.628–99999)	4.64844 (1.561–7.461)	0.00620	0.55700	0.01760	0.84400
Western-Mediterranean Group	0.00703 (0.000–0.729)	25.15625 (4.970–99999)	2.11523 (0.801–3.238)	0.00133	0.58200	0.03281	0.65100
Eastern-Mediterranean Group	0.04395 (0.000–0.698)	115.625 (9.687–99999)	2.21094 (1.041–3.016)	0.00144	0.55300	0.03706	0.59400
	Spatial Expansion						
	Parameters						
	θ (CI = 95%)	M (CI = 95%)	**τ** (CI = 95%)	SSD	P_SSD_	Raggedness	P_rag_
Eastern Group	1.26863 (0.001–3.921)	23.44725 (10.230–99999)	3.07576 (1.335–5.833)	0.00933	0.30800	0.01760	0.85800
Western-Mediterranean Group	0.02484 (0.001–1.066)	42.03757 (8.630–99999)	2.05086 (0.863–2.820)	0.00129	0.57000	0.03281	0.66500
Eastern-Mediterranean Group	0.07874 (0.001–0.958)	351.4398 (18.276–99999)	2.15680 (1.062–2.877)	0.00146	0.45200	0.03706	0.60600

Numbers in parenthesis are the upper and lower bound of 95% CI (1000 bootstrap replicates).

θ_0_ and θ_1_: pre-expansion and post-expansion populations size; τ: time in number of generations elapsed since the sudden/demographic expansion and spatial expansion episodes; SSD: sum of squared deviations; Raggedness: raggedness index following [49]; P_SSD_ and P_RAG_.: probability that expected mismatch distributions (1000 bootstrap replicates) be larger than observed mismatch distributions.

**Table 3:** Tajima's *D* and [44] Fu's *F*s test values and their statistical significance for *Philaenus spumarius* Cytochrome *c* oxidase I mtDNA groups.

	Neutrality Tests	
	Tajima's *D* test	Fu's *Fs* test
Eastern Group	0.15553	−6.28375**
Western-Mediterranean Group	−1.77941*	−23.61561***
Eastern-Mediterranean Group	−1.75709*	−26.22826***

*: indicates significant values at P<0.05; **: indicates significant values at P<0.01 and ***: indicates significant values at P<0.001.

Assuming a slight population growth for the “eastern” group, the timing of demographic expansion was estimated to have occurred at approximately 0.121 Ma ago (0.195–0.041 Ma ago), while for the “western-Mediterranean” and the “eastern-Mediterranean” haplogroups it was more recent, at 0.055 Ma ago (0.085–0.021 Ma ago) and 0.058 Ma ago (0.079–0.027 Ma ago), respectively. The spatial expansion for the three mtDNA groups was estimated to be slightly more recent than demographic expansion, at 0.080 Ma ago (0.152–0.035 Ma ago) for the “eastern” group, at 0.056 Ma ago (0.075–0.028 Ma ago) for the “eastern-Mediterranean” and at 0.054 Ma ago (0.074–0.022 Ma ago) for the “western-Mediterranean” group.

### Discussion

Biogeographical patterns, divergence time and demographic events in *Philaenus spumarius*

Our time estimates indicate that the evolutionary history of *P. spumarius* is most likely related to climate changes of the Pleistocene epoch (∼2.588–0.0117 Ma ago [1]). Divergence within species is estimated to be recent (no more than 0.5 Ma) occurring most probably in the Middle/Late Pleistocene. The biogeographical pattern of *P. spumarius* obtained from mtDNA genes shows the differentiation of two main mtDNA lineages, the “western” in the Mediterranean region and the “eastern” in Anatolia/Caucasus. Within the “western” lineage we observed two sub-lineages: the “western-Mediterranean” centred in the Iberian Peninsula and the “eastern-Mediterranean” centred in the Balkans (Fig 1). This pattern was first found by [27], who gave more emphasis to the westernmost lineages, and was later corroborated by [26] that brought some insights regarding possible refugia located in eastern Europe and western Asia. According to our data, during the Mindel or Riss glacial period, a first split between the western and eastern populations seems to have occurred with the diversification of the “western” lineage in the Mediterranean region, and of the “eastern” lineage maybe in Anatolia and surrounding area of the Caucasus, or even in territories of western Asia, as also suggested by [26].During the following interglacial, the “eastern” lineage seems to have suffered a negligible population growth compared with a more significant demographic expansion of the “western” lineage, which appears to have later retracted to two Mediterranean refugia, the Iberian Peninsula and the Balkans, where it diverged into two sub-lineages (the “western-Mediterranean” and the “eastern-Mediterranean”), maybe during the Würm glacial. After that period, the “eastern-Mediterranean” lineage centred in the Balkans seems to have expanded to the Italian Peninsula. The land bridge which existed in the northern and central part of the present Adriatic Sea between the Italian and the Balkan peninsulas, during the Quaternary cold periods [6], would have made the contact between these two peninsulas easier. The expansion was followed by a slight differentiation in Italy from the Balkans. The COI haplotype network shows that the dispersal of haplotypes from the Iberian Peninsula to the north of Italy has also occurred, maybe either crossing mountains or along the Mediterranean coast of Spain and France as suggested for *Cicada orni* [52]. Although a weak flier, it is also possible that *P. spumarius* dispersed over the sea facilitated by wind (anemohydrochoric dispersal) since this mode of dispersal has already been observed in this species [20].

The expansion dates estimated here have wide confidence intervals. However, the lowest boundaries of these dates are about 0.015 Ma ago, suggesting that the demographic and spatial expansion of this species may have occurred earlier than Holocene. Our data and reference [26] suggest the current geographic pattern of the species seems to result from postglacial range expansion from the Iberian Peninsula to the central and north-western Europe and, from the Anatolia/Caucasus (and eventually from western Asia) to east, north and central Europe, thus seemingly not following one of the common four paradigms [12], [53]. Although a northern expansion from Balkans cannot be completely ruled out, the current data indicates that the Carpathians may have represented a geographic barrier to the northern expansion of Balkans populations. Further detailed sampling and genetic analysis of the Carpathian region would be important to test this hypothesis.

Contact zones in Europe have been recorded for several European temperate species (reviewed in [54]). According to our results, the mtDNA lineages are geographically separated in most part of the range of *P. spumarius* but came into contact in some geographic regions (Fig. 1). This suggests the existence of recent admixture (secondary contact of diverged lineages) between mtDNA lineages in populations from these regions, also corroborated by [26]. The presence of the “eastern-Mediterranean” sub-lineage in some populations from the eastern part of the Iberian Peninsula (haplotype H56) and of the “western” sub-lineage in Balkans (haplotype H18) indicates that recent migrations between Mediterranean refugia may have occurred during the Quaternary period as reported in the olive fly *Bactrocera oleae* [55]. However, incomplete lineage sorting of an ancestral polymorphism cannot be ruled out as another possible explanation for the current mtDNA pattern of *P. spumarius*. Our data also indicate the existence of incomplete lineage sorting and/or admixture in the nuclear gene EF-1α. Although nuclear *clades* A and B correspond well to the “eastern-Mediterranean” and to the “western-Mediterranean” mtDNA sub-lineages, respectively, *clade* C is a mix of individuals from the three mitochondrial lineages. Heterozygous individuals whose alleles grouped within different *clades* were found, a fact not detected by [26], since they analysed homozygous individuals only. In the nuclear gene there was a lack of support for the monophyly of the “western” lineage (“western-Mediterranean and “eastern-Mediterranean” mitochondrial sub-lineages). Taken together, our results suggest that the current biogeographic pattern of *P. spumarius* may be the result of both secondary admixture and incomplete lineage sorting.

Also quite interesting is the uncommon [12] high genetic diversity detected in *P. spumarius* populations from northern Europe (Scandinavia) indicating that the north of Europe was colonized by populations that may have survived in several extra-Mediterranean glacial refugia in addition to the “classical” Mediterranean ones [7].

### Gene-flow between the Iberian Peninsula and Morocco

The presence of the “western-Mediterranean” sub-lineage in Morocco suggests a close relationship between these *P. spumarius* populations and the Iberian Peninsula. This close relationship is also corroborated by the nuclear data. There is evidence that the Strait of Gibraltar has not been an effective barrier to the dispersal, having been, in fact, the route of dispersal for many species from Africa to Europe and vice-versa [56], [57], [58]. Lowered sea levels during glacial periods possibly facilitated exchange across the Strait of Gibraltar [59]. It is quite possible that, during such lower sea level periods, individuals from the Iberian Peninsula reached North Africa via anemohydrochoric dispersal. Contrarily to the thermophilous species *Cicada barbara*, a common cicada in southern Portugal and Spain [60], the Rif Mountains did not appear to have acted as a geographical barrier to the dispersal of *P. spumarius* through Morocco, since this latter can be found in a variety of terrestrial habitats and even at altitudes above 1700m (e.g. Mt Parnassus, in Greece: observations by Ana Rodrigues, Sara Silva and Eduardo Marabuto). Although the haplotypes found in samples from Morocco were the same as some of the ones found in the Iberian Peninsula, indicating that they belong to *P. spumarius*, the analysis of four male genitalia from these populations revealed similarities with *P. tesselatus* [61] and showed the necessity of further investigation on the taxonomic status of these species, as previously suggested by [26].

### The UK and the origin of the North American and insular populations

Our analyses suggest the presence of at least two mitochondrial lineages in the UK, the “western-Mediterranean” and the “eastern”, and support a British origin for the populations of the Azores and New Zealand, and a multiple origin for the North American populations (Figs. 1 and 2). Populations from the Azores and New Zealand seem to have originated from only one of the British lineages here represented (the “western-Mediterranean” lineage). North American populations seem to have a mixed origin: a British origin, already suggested in the preliminary study by [27], from the “western-Mediterranean” lineage present in the UK, and an Iberian origin, due to the close relationship between one haplotype (H26) from the eastern United States and the Iberian Peninsula haplotypes. In fact, multiple translocations from different localities from western Europe have already been suggested for North American populations [26]. The close relationship of some North American haplotypes (New Hampshire) to the ones found in Anatolia and Finland indicates another origin from the “eastern” mitochondrial lineage, and that was never reported before. Nevertheless, a morphological variation in North American populations was already reported by [62]. The author shows that *P. spumarius* populations from New Hampshire and adjacent areas of North American exhibit morphological variation in male genitalia features and attributes this variation to hybridization between *P. spumarius* subspecies from different parts of Europe. Verification of whether there is any correlation between Hamilton's morphological subspecies and haplotype variation would require a parallel investigation of morphology beyond the scope of this work. The colonization of New Zealand and North America was probably recent and resulted from non-intentional anthropogenic introductions. This recent colonization could explain the spittlebug pest status in some locations of the USA and Canada, where *P. spumarius* reaches high densities, perhaps as result of the lack of competitors and predators [19], [24]. Concerning the Azorean populations, the fact that they only occur in high altitude native habitats (e.g. in the geologically older areas of S. Miguel), very far from human altered habitats, is somewhat puzzling and we cannot exclude the possibility of a natural colonization by long-distance dispersal.

## Conclusion

*P. spumarius* is one of the most widespread insects in Europe. We successfully provided time estimates of the main demographic and evolutionary events for the populations occurring in the main Mediterranean peninsulas, and in addition interpreted the colonization patterns out of Eurasia, namely of north-western Africa, North America, and the islands of Azores and New Zealand. This combination of a well analysed phylogeographic and demographic pattern with the multiple transcontinental colonization events, some putatively natural, others recent non-intentional anthropogenic introductions, with ecosystem level consequences, make this species well placed for understanding the long term effects of invasive species and their post-invasion evolution.

## Supporting Information

Figure S1 **Median-joining haplotype network of a set of** ***Philaenus spumarius*** **sampled geographic regions for mitochondrial gene COII (495bp).** Size of the circles is in proportion to the number of haplotypes. Branches begin in the centre of the circles and their size is in proportion to the number of mutations.
(TIF)

Figure S2 **Median-joining haplotype network of a set of** ***P. spumarius*** **sampled geographic regions for mitochondrial gene cyt** ***b*** **(434bp).** Size of the circles is in proportion to the number of haplotypes. Branches begin in the centre of the circles and their size is in proportion to the number of mutations.
(TIF)

Figure S3 **Median-joining haplotype network of** ***P. spumarius*** **sampled geographic regions for mitochondrial gene COI (289bp).** Size of the circles is in proportion to the number of haplotypes. Branches begin in the centre of the circles and their size is in proportion to the number of mutations.
(TIF)

Figure S4 **Mismatch distribution of mtDNA COI haplotypes for each of the three** ***P. spumarius*** **haplogroups.** (a) Eastern lineage; (b) Western-Mediterranean sub-lineage and (c) Eastern-Mediterranean sub-lineage. The expected frequency is based on a population growth-decline model, determined using DnaSP and is represented by a continuous line. The observed frequency is represented by a dotted line. Parameter values for the mismatch distribution are given in Table 2.
(TIF)

Figure S5 **Bayesian skyline plots showing the historical demographic trends for each main** ***Philaenus spumarius*** **mtDNA group detected using COI gene.** Along the y-axis is the expressed population size estimated in units of Neµ (Ne: effective population size, µ: mutation rate per haplotype per generation). The y-axis is in a log-scale. Solid lines represent median estimates and blue lines represent the 95% high probability density (HPD) intervals.
(TIF)

Table S1 **Analysed samples of** ***Philaenus spumarius*** **with description of the sampling locations and indication of the corresponding mtDNA Cytochrome** ***c*** **oxidase I (COI), Cytochrome** ***c*** **oxidase II (COII), Cytochrome** ***b*** **haplotype/code and Elongation Factor-1α code (EF-1α).**
(PDF)

Table S2 **Haplotype distribution within** ***P. spumarius*** **geographic regions for mitochondrial gene COI.** The total number of haplotypes per geographic region and the total number of individuals per haplotype are also shown. Western Europe: Belgium and France; Italy: Italian peninsula and Sicily.
(PDF)

Table S3 **Analyses of molecular variance (AMOVA) among regions of** ***P. spumarius*** **based on COI data.**
(PDF)

Table S4 **Divergence time estimates in million years (Ma) from the most recent common ancestor of each main** ***Philaenus spumarius*** **mtDNA COI haplogroup estimated using a mean mutation rate of 3.54% per million years as suggested by** [41] **.**
(PDF)
