# Supplementary figures and images for: New Mitochondrial and Nuclear Evidences Support Recent Demographic Expansion and an Atypical Phylogeographic Pattern in the Spittlebug Philaenus spumarius (Hemiptera, Aphrophoridae)
Source: PLoS One. 2014 Jun 3;9(6):e98375. doi: 10.1371/journal.pone.0098375 (PMC4043774; doi:10.1371/journal.pone.0098375)

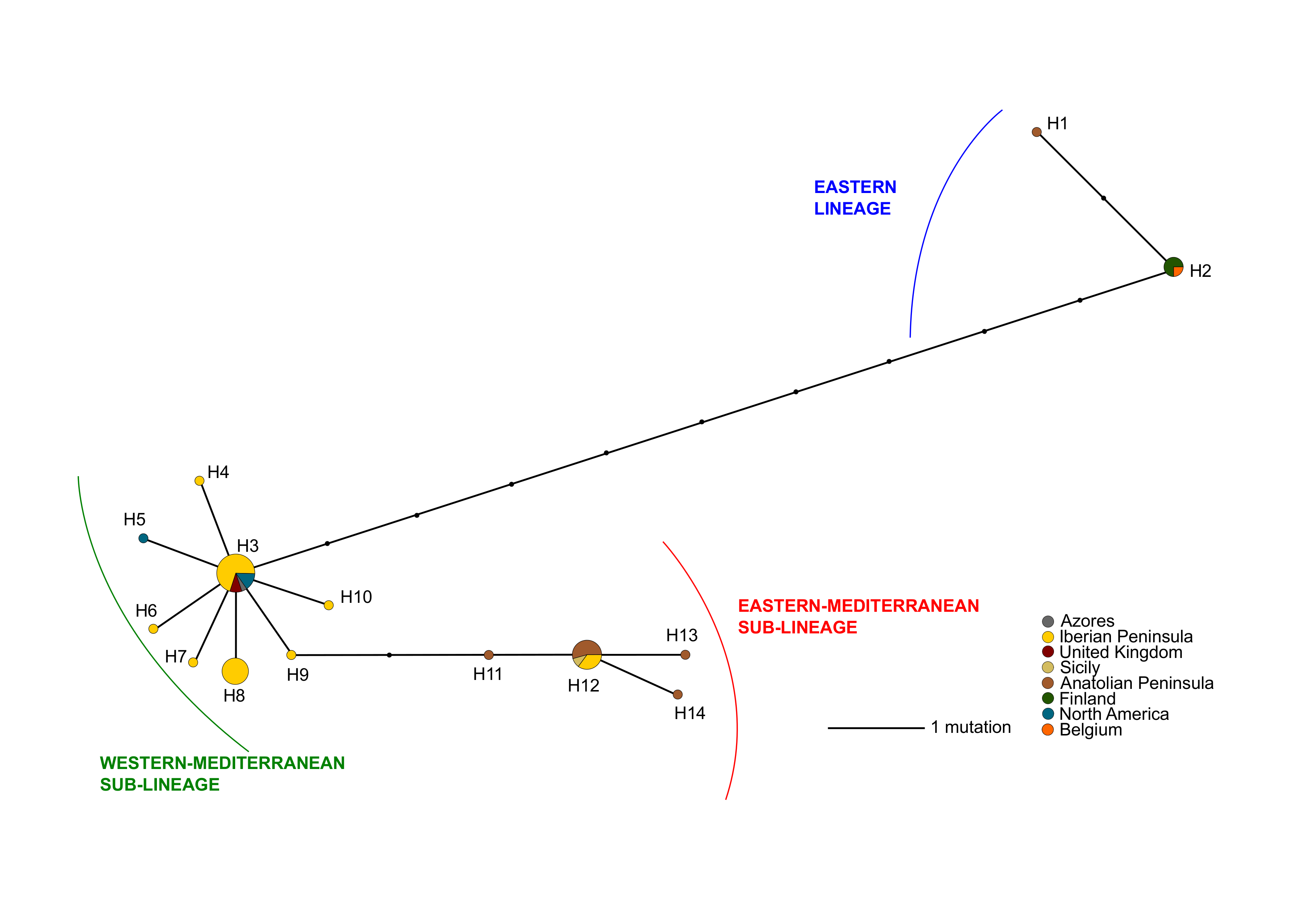

Supplement: Figure S1 — Median-joining haplotype network of a set of Philaenus spumarius sampled geographic regions for mitochondrial gene COII (495bp). Size of the circles is in proportion to the number of haplotypes. Branches begin in the centre of the circles and their size is in proportion to the number of mutations. (TIF) [file pone.0098375.s001.tif]

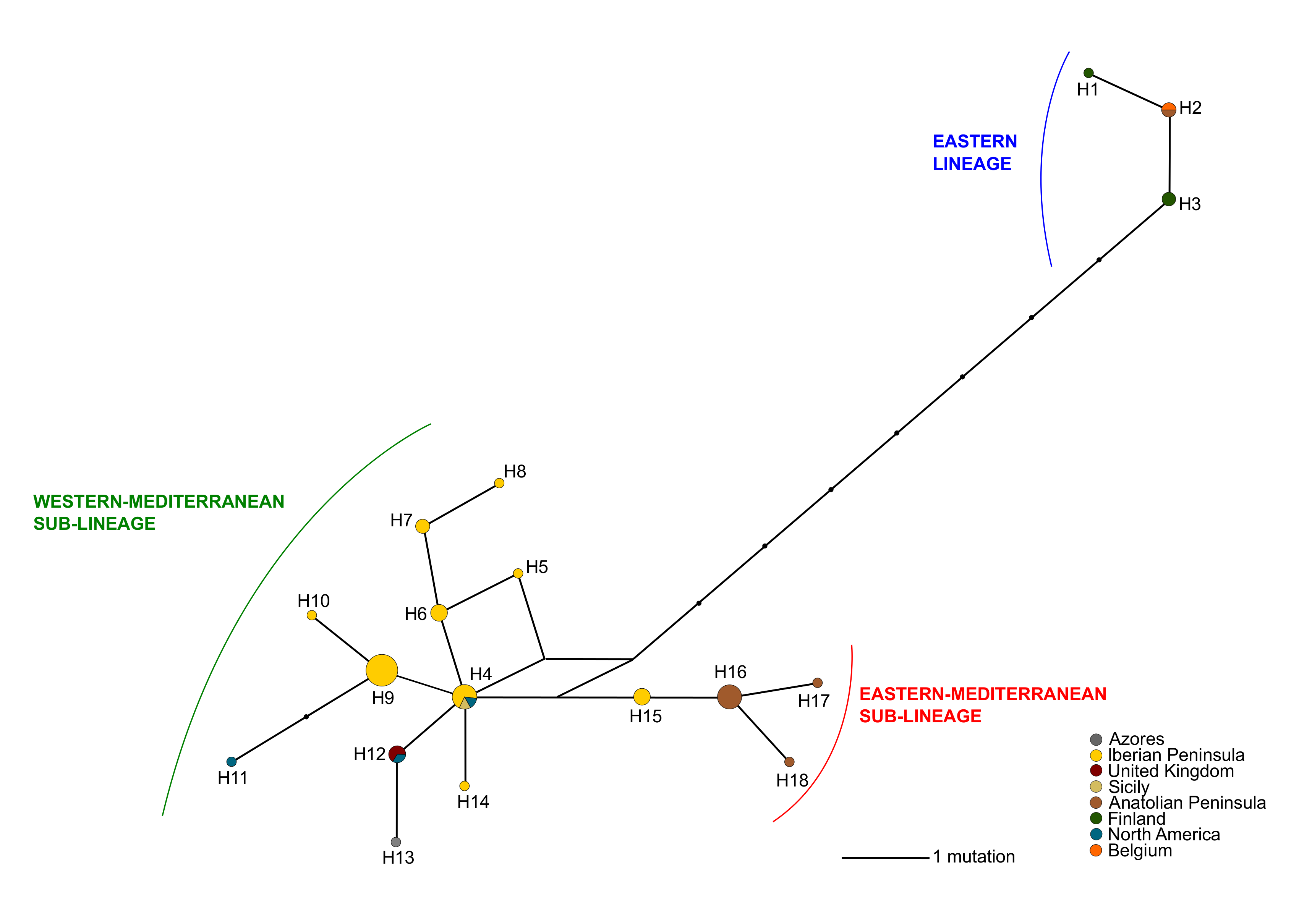

Supplement: Figure S2 — Median-joining haplotype network of a set of P. spumarius sampled geographic regions for mitochondrial gene cyt b (434bp). Size of the circles is in proportion to the number of haplotypes. Branches begin in the centre of the circles and their size is in proportion to the number of mutations. (TIF) [file pone.0098375.s002.tif]

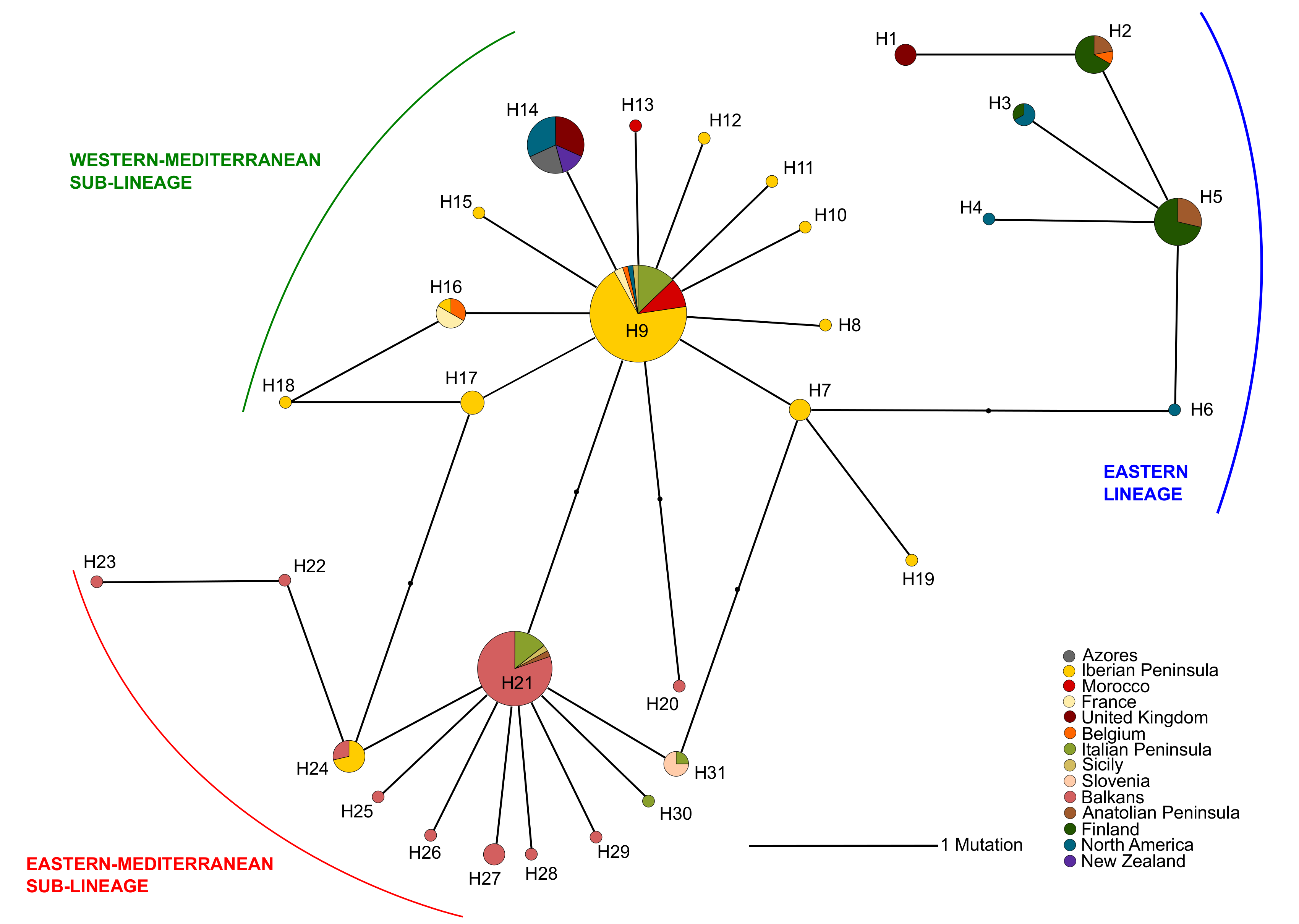

Supplement: Figure S3 — Median-joining haplotype network of P. spumarius sampled geographic regions for mitochondrial gene COI (289bp). Size of the circles is in proportion to the number of haplotypes. Branches begin in the centre of the circles and their size is in proportion to the number of mutations. (TIF) [file pone.0098375.s003.tif]

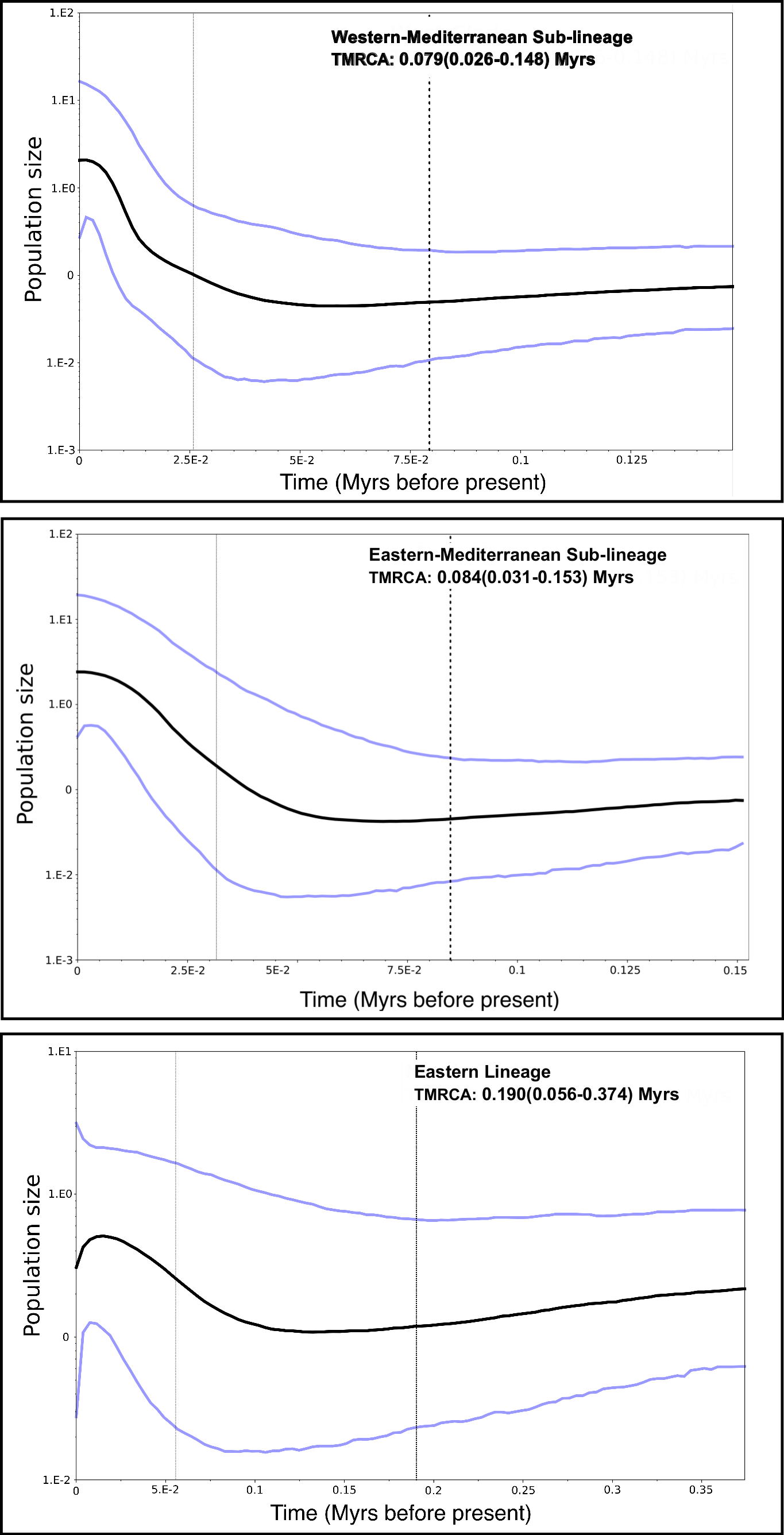

Supplement: Figure S5 — Bayesian skyline plots showing the historical demographic trends for each main Philaenus spumarius mtDNA group detected using COI gene. Along the y-axis is the expressed population size estimated in units of Neµ (Ne: effective population size, µ: mutation rate per haplotype per generation). The y-axis is in a log-scale. Solid lines represent median estimates and blue lines represent the 95% high probability density (HPD) intervals. (TIF) [file pone.0098375.s005.tif]
