# Supplementary material for: New Mitochondrial and Nuclear Evidences Support Recent Demographic Expansion and an Atypical Phylogeographic Pattern in the Spittlebug Philaenus spumarius (Hemiptera, Aphrophoridae)
Source: PLoS One. 2014 Jun 3;9(6):e98375. doi: 10.1371/journal.pone.0098375 (PMC4043774; doi:10.1371/journal.pone.0098375)
Supplement: Table S1 — Analysed samples of Philaenus spumarius with description of the sampling locations and indication of the corresponding mtDNA Cytochrome c oxidase I (COI), Cytochrome c oxidase II (COII), Cytochrome b haplotype/code and Elongation Factor-1α code (EF-1α). (PDF) [file pone.0098375.s006.pdf]

Table S1. Analysed samples of *Philaenus spumarius* with description of the sampling locations and indication of the corresponding mtDNA Cytochrome *c* oxidase I (COI), Cytochrome *c* oxidase II (COII), Cytochrome *b* haplotype/code and Elongation Factor-1α code (EF-1α).

Location (Collector)	COI Haplotype/Code	COII Haplotype/Code	Cyt *b* Haplotype/Code	EF-1α Code	GPS Coordinates
**Portugal**					
Quinta da Ranca, Vinhais, Trás-os-Montes (Seabra)	H27/Ranca_1	H7/Ranca_1	H9/Ranca_1		41°48'48.80"N; 6°59'44.51"W
Viana do Castelo, Minho (Seabra)	H30				41°42'40.10"N; 8°51'20.95"W
Parque Nacional Peneda Gerês, Minho (Rodrigues, Silva, Marabuto)	H29				41°43'44.34"N; 8° 9'46.46"W
São Jacinto, Aveiro (Seabra)	H29				40°40'36.19"N; 8°43'13.53"W
São Jacinto, Aveiro (Marabuto)	H29/S.Jacinto_3	H3/S.Jacinto_3	H9/S.Jacinto_3		40°40'36.19"N; 8°43'13.53"W
Bom Sucesso, Foz do Arelho (Quartau, Seabra & Penado)	H37				39°25'2.95"N; 9°13'39.18"W
Bom Sucesso, Foz do Arelho (Quartau, Seabra & Penado)	H36				39°25'2.95"N; 9°13'39.18"W
Bom Sucesso, Foz do Arelho (Rodrigues, Marabuto, Pereira & Seabra)	H29/ F. Arelho_8	H10/ F. Arelho_8	H9/ F. Arelho_8		39°25'2.95"N; 9°13'39.18"W
Covão da Ametade, Serra da Estrela (Seabra)	H29				40°19'33.85"N; 7°34'19.08"W
Santa Combadão, Serra da Estrela (Marabuto)	H29/S.Estrela_3	H3/S.Estrela_3	H9/S.Estrela_3		40°23'51.71"N; 8° 7'49.97"W
Olival, Ourém (Quartau)	H29				39°42'38.51"N; 8°36'4.39"W
Olival, Ourém (Quartau)	H37/ Olival_6	H9/ Olival_6	H9/ Olival_6		39°42'38.51"N; 8°36'4.39"W
Évora de Alcobaça, Alcobaça (Nunes)	H37				39°30'55.84"N; 8°58'20.40"W
Évora de Alcobaça, Alcobaça (Nunes)	H29/ Alcobaça_3	Alcobaça_3	Alcobaça_3		39°30'55.84"N; 8°58'20.40"W
Évora de Alcobaça, Alcobaça (Nunes)	H37/ Alcobaça_4	H3/ Alcobaça_4	H9/ Alcobaça_4		39°30'55.84"N; 8°58'20.40"W
Évora de Alcobaça, Alcobaça (Nunes)	H29				39°30'55.84"N; 8°58'20.40"W
Serra d' Aire (Seabra)	H21/S.Aire_5	H4/S.Aire_5	H9/S.Aire_5		39°31'56.98"N; 8°31'52.56"W
Serra d' Aire (Seabra)	H37				39°31'56.98"N; 8°31'52.56"W
Serra Montejunto (Marabuto)	H29/Montejunto_2	H3/Montejunto_2	H9/Montejunto_2		39°10'24.53"N; 9° 2'55.66"W
Gouveia, Sintra (Quartau, Seabra & Penado)	H36				38°50'15.75"N; 9°25'20.77"W
Fontanelas, Sintra (Rodrigues, Seabra & Pereira)	H29/ Sintra_10	H3/ Sintra_10	H9/ Sintra_10	Sintra_10	38°50'15.75"N; 9°25'20.77"W
Arrábida, Setúbal (Fonseca)	H29			Arrabida_5	38°28'20.09"N; 8°59'41.77"W
Arrábida, Setúbal (Fonseca)	H37/Arrabida_6	H8/Arrabida_6	H6/Arrabida_6		38°28'20.09"N; 8°59'41.77"W
Vale do Gaio, Torrão, Alentejo (Seabra)	H37				38°14'57.66"N; 8°17'49.32"W
Grândola, Alentejo (Seabra)	H29				38°10'5.83"N; 8°36'34.40"W
Grândola, Alentejo (Seabra)	H22				38°10'5.83"N; 8°36'34.40"W
Grândola, Alentejo (Quartau & Seabra)	H38/Grandola_1	H8/Grandola_1	H6/Grandola_1		38°10'5.83"N; 8°36'34.40"W
Grândola, Alentejo (Quartau & Seabra)	H26				38°10'5.83"N; 8°36'34.40"W
Beringel, Ferreira do Alentejo (Quartau & Simões)	H29/F.Alentejo_1	H3/F.Alentejo_1	H4/F.Alentejo_1		38° 3'23.09"N; 7°59'5.45"W
Beja, Alentejo (Marabuto)	H37				38° 5'22.11"N; 7°58'26.53"W
Sines, Alentejo (Rodrigues & Marabuto)	H29/Sines_1	H3/Sines_1	H4/Sines_1		37°57'58.98"N; 8°52'25.14"W
Cercal, Alentejo (Seabra)	H40				37°46'39.12"N; 8°39'46.80"W
Ribeira do Torgal, Odemira, Alentejo (Ribeiro & Pires)	H20				37°39'35.14"N; 8°37'40.86"W
Ribeira do Torgal, Odemira, Alentejo (Ribeiro & Pires)	H29				37°39'35.14"N; 8°37'40.86"W
Ribeira do Torgal, Odemira, Alentejo (Ribeiro & Pires)	H29				37°39'35.14"N; 8°37'40.86"W
Santa Clara a Velha, Rio Mira, Alentejo (Ribeiro)	H40/R.Mira_1	H8/R.Mira_1	H8/R.Mira_1		37°30'38.22"N; 8°28'27.23"W
Santa Clara a Velha, Rio Mira, Alentejo (Ribeiro)	H39/Mira_1	H8/Mira_1	H6/Mira_1		37°30'38.22"N; 8°28'27.23"W
Santa Clara a Velha, Rio Mira, Alentejo (Ribeiro)	H40/Mira_2	H8/Mira_2	H7/Mira_2		37°30'38.22"N; 8°28'27.23"W
Santa Clara a Velha, Rio Mira, Alentejo (Rodrigues & Marabuto)	H29/Mira_3	H3/Mira_3	H4/Mira_3		37°30'38.22"N; 8°28'27.23"W
Santa Clara a Velha, Rio Mira, Alentejo (Rodrigues & Marabuto)	H37	H8	H5		37°30'38.22"N; 8°28'27.23"W
Aljezur, Alentejo (Rodrigues & Marabuto)	H41/Aljejur_9.1	H8/Aljejur_9.1	H7/Aljejur_9.1		37°18'11.22"N; 8°47'59.88"W
Silves, Algarve (Seabra)	H29				37°11'40.31"N; 8°27'55.28"W
Silves, Algarve (Seabra)	H29				37°11'40.31"N; 8°27'55.28"W

Location (Collector)	COI Haplotype	COII Haplotype	Cyt *b* Haplotype	EF code	GPS Coordinates
**Portugal**					
Silves, Algarve (Seabra)	H29/Silves_3	H3/Silves_3	Silves_3		37°11'40.31"N; 8°27'55.28"W
Silves, Algarve (Seabra)	H29				37°11'40.31"N; 8°27'55.28"W
Silves, Algarve (Seabra)	H29				37°11'40.31"N; 8°27'55.28"W
Silves, Algarve (Seabra)	H29				37°11'40.31"N; 8°27'55.28"W
Barranco do Velho, Algarve (Quartau)	H29				37° 8'15.13"N; 8° 1'24.27"W
São Miguel, Açores (Borges)	H24/ Azores_1	H3/ Azores_1	H13/ Azores_1	Azores_1	37°47'46.86"N; 25°11'4.50"W
São Miguel, Açores (Borges)	H24				37°47'46.86"N; 25°11'4.50"W
São Miguel, Açores (Borges)	H71				37°47'46.86"N; 25°11'4.50"W
São Miguel, Açores (Borges)	H24				37°47'46.86"N; 25°11'4.50"W
São Miguel, Açores (Borges)	H24				37°47'46.86"N; 25°11'4.50"W
**Finland**					
Tvarminne (Halkka)	H2/ Tvarminne_4	H2/ Tvarminne_4	H1/ Tvarminne_4		59°50'37.60"N; 23°14'21.68"E
Tvarminne (Halkka)	H16				59°50'37.60"N; 23°14'21.68"E
Tvarminne (Halkka)	H16				59°50'37.60"N; 23°14'21.68"E
Norra Grisselgrundet (Halkka)	Grisselgrundet_4	Grisselgrundet_4	Grisselgrundet_4		59°50'17.48"N; 23°14'46.87"E
Norra Grisselgrundet (Halkka)	H16				59°50'17.48"N; 23°14'46.87"E
Windskar (Halkka)	H2				59°49'40.56"N; 23°12'42.74"E
Brannskar (Halkka)	H5/ Brannskar_7	H2/ Brannskar_7	H3/ Brannskar_7		59°50'39.51"N; 23°16'22.50"E
Brannskar (Halkka)	H15				59°50'39.51"N; 23°16'22.50"E
Brannskar (Halkka)	H15				59°50'39.51"N; 23°16'22.50"E
Brannskar (Halkka)	H16				59°50'39.51"N; 23°16'22.50"E
Segelskar (Halkka)	H8/ Segelskar_4	H2/ Segelskar_4	H3/ Segelskar_4		59°45'52.22"N; 23°22'24.53"E
Segelskar (Halkka)	H16				59°45'52.22"N; 23°22'24.53"E
Punkalaidun (Halkka)	H1				61° 7'0.75"N; 23° 5'35.54"E
Punkalaidun (Halkka)	H4				61° 7'0.75"N; 23° 5'35.54"E
Punkalaidun (Halkka)	H7				61° 7'0.75"N; 23° 5'35.54"E
Punkalaidun (Halkka)	H12				61° 7'0.75"N; 23° 5'35.54"E
Haaparnaki-Keuruu (Halkka)	H4				62°15'32.75"N; 24°42'28.08"E
Haaparnaki-Keuruu (Halkka)	H7				62°15'32.75"N; 24°42'28.08"E
**Turkey**					
Cerkes (Yurtserver)	Cerkes_3	H1/ Cerkes_3	H2/ Cerkes_3		40°48'59.24"N; 32°54'9.50"E
Cerkes (Yurtserver)	H4				40°48'59.24"N; 32°54'9.50"E
Cerkes (Yurtserver)	H6				40°48'59.24"N; 32°54'9.50"E
Cerkes (Yurtserver)	H6				40°48'59.24"N; 32°54'9.50"E
Cerkes (Yurtserver)	H11				40°48'59.24"N; 32°54'9.50"E
Cerkes (Yurtserver)	H14			Turkey_3	40°48'59.24"N; 32°54'9.50"E
Cerkes (Yurtserver)	H14				40°48'59.24"N; 32°54'9.50"E
Azdavay (Yurtserver)	H57/Azdavay_1	H11/Azdavay_1	H16/Azdavay_1		41°38'29.26"N; 33°17'52.54"E
Keçan (North) (Yurtserver)	H57/ Keçan_N1	H13/Keçan_N1	H17/Keçan_N1	Keçan_N1	40°53'29.60"N; 26°38'42.81"E
Keçan (North) (Yurtserver)	H64				40°53'29.60"N; 26°38'42.81"E
Keçan (South) (Yurtserver)	H57/Keçan_S1	H12/Keçan_S1	H16/Keçan_S1		40°44'41.83"N; 26°36'6.98"E
Keçan (South) (Yurtserver)	H64				40°44'41.83"N; 26°36'6.98"E
Lapseki (Yurtserver)	H46/ Lapseki_1	H12/ Lapseki_1	H16/ Lapseki_1		40°19'3.46"N; 26°43'46.86"E
Lapseki (Yurtserver)	H57				40°19'3.46"N; 26°43'46.86"E
Lapseki (Yurtserver)	H59				40°19'3.46"N; 26°43'46.86"E
Kucukuyu (Yurtserver)	H55/ Kucukuyu_1	H12/ Kucukuyu_1	H16/ Kucukuyu_1		39°36'35.48"N; 26°33'2.11"E
Kucukuyu (Yurtserver)	H57				39°36'35.48"N; 26°33'2.11"E
Kucukuyu (Yurtserver)	H67				39°36'35.48"N; 26°33'2.11"E
Suloglu (Yurtserver)	H57/ Suloglu_1	H14/ Suloglu_1	H16/ Suloglu_1		41°46'52.25"N; 26°53'1.74"E

Location (Collector)	COI Haplotype	COII Haplotype	Cyt *b* Haplotype	EF code	GPS Coordinates
**Turkey**					
Suloglu (Yurtserver)	H57				41°46'52.25"N; 26°53'1.74"E
Suloglu (Yurtserver)	H62				41°46'52.25"N; 26°53'1.74"E
Pabucdere (Yurtserver)	H50/ Pabucdere_1	H14/ Pabucdere_1	H16/ Pabucdere_1		41°38'18.49"N; 27°54'54.61"E
Pabucdere (Yurtserver)	H57				41°38'18.49"N; 27°54'54.61"E
Pabucdere (Yurtserver)	H64				41°38'18.49"N; 27°54'54.61"E
Demirkoy (Yurtserver)	H54/ Demirkoy_1	H12/ Demirkoy_1	H18/ Demirkoy_1		41°52'34.74"N; 27°46'8.04"E
Demirkoy (Yurtserver)	H57				41°52'34.74"N; 27°46'8.04"E
**Italy**					
Tardaria, Etna, Sicily (d'Urso)			H4		37°43'53.05"N; 14°59'8.12"E
Tardaria, Etna, Sicily (d'Urso)	H29				37°43'53.05"N; 14°59'8.12"E
Bosco di Acisantantonio, Etna, Sicily (d'Urso)	H49/Sicily_5	H12/Sicily_5	Sicily_5		37°41'16.21"N; 15° 9'30.01"E
Torcello, Veneze (Quartau)	H47				45°29'47.21"N; 12°25'11.44"E
Via Appia, Rome (Quartau)	H53				41°44'33.26"N; 12°42'44.66"E
Emilia, Parma (Quartau)	H32				44°48'12.33"N; 10°20'45.45"E
Emilia, Parma (Quartau)	H32				44°48'12.33"N; 10°20'45.45"E
Emilia, Parma (Quartau)	H32				44°48'12.33"N; 10°20'45.45"E
Emilia, Parma (Quartau)	H32				44°48'12.33"N; 10°20'45.45"E
Emilia, Parma (Quartau)	H32				44°48'12.33"N; 10°20'45.45"E
Emilia, Parma (Quartau)	H32				44°48'12.33"N; 10°20'45.45"E
Emilia, Parma (Quartau)	H33			Italia_2	44°48'12.33"N; 10°20'45.45"E
Emilia, Parma (Quartau)	H51				44°48'12.33"N; 10°20'45.45"E
Florence, Toscana (Marabuto)	H49				43°49'21.87"N; 11°21'9.66"E
Mt. Calvi, Toscana (Marabuto)	H53				43° 5'9.00"N; 10°36'28.00"E
Greve in Chianti, Toscana (Marabuto)	H49				43°35'2.00"N; 11°18'50.00"E
Bologna (Marabuto)	H52				44° 2'45.00"N; 11°17'44.00"E
Villaromagnana, Alps (Lessio)	H32				44°50'59.56"N; 8°53'25.52"E
**United Kingdom**					
Cardiff, Wales (Wilson)	H24				51°28'57.16"N; 3°11'0.41"W
Cardiff, Wales (Wilson)	H24				51°28'57.16"N; 3°11'0.41"W
Aberdare, Wales (Wilson)	H13			UK_7	51°42'49.13"N; 3°26'43.27"W
Aberdare, Wales (Wilson)	H13				51°42'49.13"N; 3°26'43.27"W
Aberdare, Wales (Wilson)	H13				51°42'49.13"N; 3°26'43.27"W
Aberdare, Wales (Wilson)	H24				51°42'49.13"N; 3°26'43.27"W
Cambridge, England (Borges)	H23/Cambridge_1	H3/Cambridge_1	H12/Cambridge_1		52°12'22.80"N; 0° 7'29.41"E
Cambridge, England (Borges)	H23/Cambridge_2	H3/Cambridge_2	H12/Cambridge_2		52°12'22.80"N; 0° 7'29.41"E
Cambridge, England (Borges)	H24				52°12'22.80"N; 0° 7'29.41"E
Oxfordshire, England (Corlev)	H24				51°39'30.43"N; 1°35'5.11"W
**Canada**					
Burnaby Mt. British Columbia (Beckenbach)	H24				53°43'36.00"N; 127°38'51.43"W
Burnaby Mt. British Columbia (Beckenbach)	H24/Canada_3	H3/Canada_3	H12/Canada_3		53°43'36.00"N; 127°38'51.43"W
Burnaby Mt. British Columbia (Beckenbach)	H70				53°43'36.00"N; 127°38'51.43"W
Burnaby Mt. British Columbia (Beckenbach)	Canada_5	Canada_5	Canada_5		53°43'36.00"N; 127°38'51.43"W
**United States of America**					
Michigan (Fonseca)	H25			USA_2	44°18'49.13"N; 85°35'6.26"W
Michigan (Fonseca)	H25				44°18'49.13"N; 85°35'6.26"W
Michigan (Fonseca)	H25/USA_4	H5/USA_4	H4/USA_4		44°18'49.13"N; 85°35'6.26"W
Michigan (Fonseca)	H25				44°18'49.13"N; 85°35'6.26"W
Michigan (Fonseca)	H26/USA_6	H3/USA_6	H11/USA_6		44°18'49.13"N; 85°35'6.26"W
Wonalancet – New Hampshire (Thompson)	H1				43°53'58.93"N; 71°21'33.47"W
Wonalancet – New Hampshire (Thompson)	H3				43°53'58.93"N; 71°21'33.47"W

Location (Collector)	COI Haplotype	COII Haplotype	Cyt *b* Haplotype	EF code	GPS Coordinates
**United States of America**					
Wonalancet – New Hampshire (Thompson)	H9				43°53'58.93"N; 71°21'33.47"W
Wonalancet – New Hampshire (Thompson)	H10				43°53'58.93"N; 71°21'33.47"W
**Spain**					
C. R. Fauna et Ed. Amb. "Los Hornos" (Rodrigues, Silva, Marabuto, Nunes & Ferreira)	H30				39°26'14.10"N; 6°17'17.46"W
Valdenoches, Castille La Mancha (Rodrigues, Silva, Marabuto, Nunes & Ferreira)	H56/Spain_3.1	H12/Spain_3.1	H15/Spain_3.1		40°41'11.03"N; 3° 5'18.87"W
Caspe, Aragon (Rodrigues, Silva, Marabuto, Nunes & Ferreira)	H56/Spain_4.1	H12/Spain_4.1	H15/Spain_4.1		41°21'41.30"N; 0° 6'17.83"W
Tolva, Aragon (Rodrigues, Silva, Marabuto, Nunes & Ferreira)	H26				42° 6'42.89"N; 42° 6'42.89"N
El Pont de Suert, Catalunha (Rodrigues, Silva, Marabuto, Nunes & Ferreira)	H35				42°24'46.98"N; 0°44'22.89"E
Forgais de Montclús, Catalunha (Rodrigues, Silva, Marabuto, Nunes & Ferreira)	H19				41°43'42.20"N; 2°26'13.21"E
Chert, Valencia (Rodrigues, Silva, Marabuto, Nunes & Ferreira)	H56				40°30'55.36"N; 0° 8'27.38"E
San Agustin, Aragon (Rodrigues, Silva, Marabuto, Nunes & Ferreira)	H56				40° 8'24.18"N; 0°43'0.50"W
Cheste, Valencia (Rodrigues, Silva, Marabuto, Nunes & Ferreira)	H56/Spain_22.1	H12/Spain_22.1	H15/Spain_22.1		39°31'9.33"N; 0°40'8.49"W
Vélez-Rubio, Andalusia (Rodrigues, Silva, Marabuto, Nunes & Ferreira)	H29				37°38'49.42"N; 2° 5'13.65"W
Ronda, Andalusia (Rodrigues, Silva, Marabuto, Nunes & Ferreira)	H29				36°43'52.11"N; 5°10'55.79"W
El Gastor, Andalusia (Rodrigues, Silva, Marabuto, Nunes & Ferreira)	H29				36°50'16.20"N; 5°20'51.02"W
Valdés, Asturias (Rodrigues, Silva & Nunes)	H34/Spain_1	H3/Spain_1	H10/Spain_1		43°32'49.08"N; 6°31'28.92"W
Meira, Galiza (Rodrigues, Silva & Nunes)	H34/Spain_2	H3/Spain_2	H9/Spain_2	Spain_2	43°13'44.40"N; 7°17'26.64"W
Aracena, Anadalusia (Marabuto)	H42				37°53'39.42"N; 6°33'43.16"W
**France**					
Saint-Antonin-Noble-Val (Rodrigues, Silva, Marabuto, Nunes & Ferreira)	H19				44° 9'29.52"N; 1°43'16.45"E
Lautrec (Rodrigues, Silva, Marabuto, Nunes & Ferreira)	H29				43°42'47.14"N; 2° 7'6.04"E
Fitou (Rodrigues, Silva, Marabuto, Nunes & Ferreira)	H19				42°52'49.11"N; 2°59'34.12"E
Aube, Lusigny-sur-Barse (Constant)	H17				48°15'7.19"N; 4°16'7.76"E
Aube, Lusigny-sur-Barse (Constant)	H29				48°15'7.19"N; 4°16'7.76"E
**Belgium**					
Namur (Constant)	H16/ Belgium_1	H2/ Belgium_1	H2/ Belgium_1	Belgium_1	50°27'55.51"N; 4°52'3.33"E
Namur (Constant)	H19				50°27'55.51"N; 4°52'3.33"E
Namur (Constant)	H19				50°27'55.51"N; 4°52'3.33"E
Namur (Constant)	H29			Belgium_4	50°27'55.51"N; 4°52'3.33"E
**Morocco**					
Near Rabat (Rodrigues, Silva, Marabuto & Ferreira)	H28				33°46'37.56"N; 7°13'58.92"W
Rabat (Rodrigues, Silva, Marabuto & Ferreira)	H29				34° 0'41.70"N; 6°42'32.94"W
Rif (Rodrigues, Silva, Marabuto & Ferreira)	H29			Morocco_6.1	35°51'58.14"N; 5°24'30.30"W
Ifrane (Rodrigues, Silva, Marabuto & Ferreira)	H37				33°30'41.82"N; 5° 5'34.08"W
Ifrane Centre (Rodrigues, Silva, Marabuto & Ferreira)	H37				33°31'58.86"N; 5° 6'7.86"W
Azrou (Rodrigues, Silva, Marabuto & Ferreira)	H29				33°26'57.78"N; 5°13'55.14"W
Azrou (Rodrigues, Silva, Marabuto & Ferreira)	H29				33°29'39.48"N; 5°15'47.46"W
**Greece**					
Bralou (Rodrigues, Silva, Marabuto)	H57				38°44'35.70"N; 22°26'55.50"E
Iti National Park (Rodrigues, Silva, Marabuto)	H63				38°44'6.96"N; 22°22'10.20"E
Mt Olympus (Rodrigues, Silva, Marabuto)	H43				40° 6'22.32"N; 22°27'33.66"E
Mt Olympus (Rodrigues, Silva, Marabuto)	H68				40° 5'35.94"N; 22°25'10.62"E
Mt Olympus (Rodrigues, Silva, Marabuto)	H18				40° 5'3.12"N; 22°24'25.26"E
Meteora (Rodrigues, Silva, Marabuto)	H57				39°43'11.76"N; 21°38'14.28"E
Mt Vourinos (Rodrigues, Silva, Marabuto)	H66				40° 6'28.50"N; 21°40'21.78"E
Mt Vourinos (Rodrigues, Silva, Marabuto)	H57				40° 9'26.94"N; 21°43'36.36"E
Mt Vourinos (Rodrigues, Silva, Marabuto)	H65				40°12'1.68"N; 21°39'30.00"E
Mt Giona (Rodrigues, Silva, Marabuto)	H57				38°40'29.40"N; 22°18'31.62"E
Mt Giona (Rodrigues, Silva, Marabuto)	H44				38°40'6.24"N; 22°18'31.62"E
Mt Parnassus (Rodrigues, Silva, Marabuto)	H57				38°36'50.16"N; 22°34'33.00"E

**Location (Collector)**	**COI Haplotype**	**COII Haplotype**	**Cyt *b* Haplotype**	**EF code**	**GPS Coordinates**
**Greece**					
Mt Parnassus (Rodrigues, Silva, Marabuto)	H46				38°34'42.24"N; 22°34'30.30"E
Mt Parnassus (Rodrigues, Silva, Marabuto)	H57				38°37'23.94"N; 22°33'12.96"E
Mt Parnassus (Rodrigues, Silva, Marabuto)	H69				38°33'19.98"N; 22°34'44.04"E
Mt Menalo (Rodrigues, Silva, Marabuto)	H45				37°37'50.64"N; 22°19'34.56"E
Mt Menalo (Rodrigues, Silva, Marabuto)	H57				37°38'26.10"N; 22°16'1.62"E
Mt Menalo (Rodrigues, Silva, Marabuto)	H60				37°40'49.38"N; 22°13'46.26"E
Mt Menalo (Rodrigues, Silva, Marabuto)	H58				37°37'37.32"N; 22°17'39.18"E
Mt Taygetus (Rodrigues, Silva, Marabuto)	H61				36°53'20.10"N; 22°21'4.68"E
Mt Parnonas (Rodrigues, Silva, Marabuto)	H57				37°11'13.44"N; 22°33'30.96"E
**Slovenia**					
Dragonja (Derlink)	H48			Slovenia_1	45°27'18.00"N; 13°42'4.68"E
Dragonja (Derlink)	H48				45°27'18.00"N; 13°42'4.68"E
Gorice pri Famljah (Derlink)	H48				45°40'21.05"N; 14° 0'52.99"E
**Bulgaria**					
Petrich (Paulo)	H57				41°25'60.00"N; 23° 1'0.00"E
**New Zealand**					
Lincoln, South Island (Yurtserver)	H14 FigS3				43°38'40.41"S; 172°28'9.98"E
Lincoln, South Island (Yurtserver)	H14 FigS3				43°38'40.41"S; 172°28'9.98"E
Lincoln, South Island (Yurtserver)	H14 FigS3				43°38'40.41"S; 172°28'9.98"E
