# Supplementary material for: New Mitochondrial and Nuclear Evidences Support Recent Demographic Expansion and an Atypical Phylogeographic Pattern in the Spittlebug Philaenus spumarius (Hemiptera, Aphrophoridae)
Source: PLoS One. 2014 Jun 3;9(6):e98375. doi: 10.1371/journal.pone.0098375 (PMC4043774; doi:10.1371/journal.pone.0098375)
Supplement: Table S3 — Analyses of molecular variance (AMOVA) among regions of P. spumarius based on COI data. (PDF) [file pone.0098375.s008.pdf]

Table S3. Analyses of molecular variance (AMOVA) among regions of *P. spumarius* based on COI data.

Source of variation	d.f.	Sum of squares	Variance of components	% of variation
**[North America] [Azores] [North Africa] [Northern Europe] [Western Europe] [South-western Europe] [Central Europe] [South-eastern Europe] [South-western Asia]*				
Among groups	8	196.948	0.73116 Va	27.73
Among regions within groups	4	29.265	0.66613 Vb	25.26
Within regions	177	219.194	1.23930 Vc	**47.00**
**Total**	189	445.568	2.63658	
***[North America] [Azores] [North Africa] [Northern Europe] [United Kingdom] [Southern Europe] [Central Europe] [South-western Asia]*				
Among groups	7	155.904	0.77953 Va	27.59
Among regions within groups	5	70.309	0.80628 Vb	28.54
Within regions	177	219.356	1.23930 Vc	**43.87**
**Total**	189	445.568	2.82511	

* ***North America:*** United States of America and Canada; ***North Africa:*** Morocco; ***Northern Europe:*** Finland; ***Western Europe:*** United Kingdom, France and Belgium; ***South-western Europe:*** Iberian Peninsula; ***Central Europe:*** Slovenia; ***South-eastern Europe:*** Italian Peninsula, Sicily and Balkans; ***South-western Asia:*** Anatolia Peninsula.

** ***North America:*** United States of America and Canada; ***North Africa:*** Morocco; ***Northern Europe:*** Finland; ***Southern Europe:*** Iberian Peninsula, Italian Peninsula, Sicily and Balkans; ***Central Europe:*** Belgium, France and Slovenia; ***South-western Asia:*** Anatolia Peninsula.
