# Supplementary material for: New Mitochondrial and Nuclear Evidences Support Recent Demographic Expansion and an Atypical Phylogeographic Pattern in the Spittlebug Philaenus spumarius (Hemiptera, Aphrophoridae)
Source: PLoS One. 2014 Jun 3;9(6):e98375. doi: 10.1371/journal.pone.0098375 (PMC4043774; doi:10.1371/journal.pone.0098375)
Supplement: Table S4 — Divergence time estimates in million years (Ma) from the most recent common ancestor of each main Philaenus spumarius mtDNA COI haplogroup estimated using a mean mutation rate of 3.54% per million years as suggested by [41] . (PDF) [file pone.0098375.s009.pdf]

Table S4. Divergence time estimates in million years (Ma) from the most recent common ancestor of each main *Philaenus spumarius* mtDNA COI haplogroup estimated using a mean mutation rate of 3.54% per million years as suggested by [41].

	Lower 95% HPD	Mean	Upper 95% HPD
Western-Mediterranean	0.026	0.079	0.148
Eastern-Mediterranean	0.031	0.084	0.153
Eastern	0.056	0.190	0.374
Eastern vs western-Mediterranean	0.118	0.269	0.450
Eastern vs eastern-Mediterranean	0.120	0.270	0.447
Eastern-Mediterranean vs western-Mediterranean	0.067	0.146	0.243
Combined	0.122	0.270	0.448
