# Supplementary material for: New Mitochondrial and Nuclear Evidences Support Recent Demographic Expansion and an Atypical Phylogeographic Pattern in the Spittlebug Philaenus spumarius (Hemiptera, Aphrophoridae)
Source: PLoS One. 2014 Jun 3;9(6):e98375. doi: 10.1371/journal.pone.0098375 (PMC4043774; doi:10.1371/journal.pone.0098375)
Supplement: Table S2 — Haplotype distribution within P. spumarius geographic regions for mitochondrial gene COI. The total number of haplotypes per geographic region and the total number of individuals per haplotype are also shown. Western Europe: Belgium and France; Italy: Italian peninsula and Sicily. (PDF) [file pone.0098375.s007.pdf]

Table S2. Haplotype distribution within *P. spumarius* geographic regions for mitochondrial gene COI. The total number of haplotypes per geographic region and the total number of individuals per haplotype are also shown. Western Europe: Belgium and France; Italy: Italian peninsula and Sicily.

Geographic Regions	Haplotypes																																		
	1	2	3	4	5	6	7	8	9	10	11	12	13	14	15	16	17	18	19	20	21	22	23	24	25	26	27	28	29	30	31	32	33	34	35	36
Azores																								4												
Iberian Peninsula																			1	1	1	1				2	1		28	1	1			2	1	2
Morocco																												1	4							
Western Europe																1	1		4										3							
United Kingdom													3										2	5												
Italy																													1			7	1			
Slovenia																																				
Balkans																		1																		
Anatolian Peninsula				1		2					1			2																						
Finland	1	2		2	1		2	1				1			2	5																				
North America	1		1						1	1														2	4	1										
Total	2	2	1	3	1	2	2	1	1	1	1	1	3	2	2	6	1	1	5	1	1	1	2	11	4	3	1	1	36	1	1	7	1	2	1	2

Geographic Regions	Haplotypes																																		
	37	38	39	40	41	42	43	44	45	46	47	48	49	50	51	52	53	54	55	56	57	58	59	60	61	62	63	64	65	66	67	68	69	70	71	Total
Azores																																			1	2
Iberian Peninsula	9	1	1	3	1	1														5																19
Morocco	2																																			3
Western Europe																																				4
United Kingdom																																				3
Italy											1		3		1	1	2																			8
Slovenia												3																								1
Balkans							1	1	1	2				1				1	1		17	1	1	1	1	1	1	3	1	1	1	1	1			21
Anatolian Peninsula																					1															5
Finland																																				9
North America																																		1		8
Total	11	1	1	3	1	1	1	1	1	2	1	3	3	1	1	1	2	1	1	5	18	1	1	1	1	1	1	3	1	1	1	1	1	1	1	
